# *Melissococcus plutonius* Can Be Effectively and Economically Detected Using Hive Debris and Conventional PCR

**DOI:** 10.3390/insects12020150

**Published:** 2021-02-09

**Authors:** Jana Biová, Jean-Daniel Charrière, Silvie Dostálková, Mária Škrabišová, Marek Petřivalský, Jaroslav Bzdil, Jiří Danihlík

**Affiliations:** 1 Department of Biochemistry, Faculty of Science, Palacký University Olomouc, Šlechtitelů 27, 783 71 Olomouc, Czech Republic; jana.biova@upol.cz (J.B.); silvie.dostalkova@upol.cz (S.D.); maria.skrabisova@upol.cz (M.Š.); marek.petrivalsky@upol.cz (M.P.); 2 Agroscope, Swiss Bee Research Center, Schwarzenburgstraße 161, 3003 Bern, Switzerland; jean-daniel.charriere@agroscope.admin.ch; 3 State Veterinary Institute, Jakoubka ze Stříbra 1, 779 00 Olomouc, Czech Republic; vetmed@seznam.cz

**Keywords:** European foulbrood, hive debris, honey bee, *Melissococcus plutonius*, PCR

## Abstract

**Simple Summary:**

Laboratory diagnostics of the presence of *Melissococcus plutonius* is necessary for the confirmation of European foulbrood (EFB) in honey bee colonies. Seeking EFB positive colonies is based on inspections performed by beekeepers or authorized inspectors. Bee brood, adult bees, or honey are usually sampled and confirmed by PCR. Here, we tested a new concept of searching suspected colonies by simple and effective collecting hive debris. Conventional PCR detection of *M. plutonius* in hive debris gives comparable sensitivity and specificity as for adults or honey. The main advantage of testing hive debris combines its effectiveness and costs compared to the sampling of adult bees or honey. Easy and fast collection of hive debris samples in areas with EFB outbreaks could fasten disease early detection and elimination.

**Abstract:**

European foulbrood (EFB) is an infectious disease of honey bees caused by the bacterium *Melissococcus plutonius*. A method for DNA isolation and conventional PCR diagnosis was developed using hive debris, which was non-invasively collected on paper sheets placed on the bottom boards of hives. Field trials utilized 23 honey bee colonies with clinically positive symptoms and 21 colonies without symptoms. Bayes statistics were applied to calculate the comparable parameters for EFB diagnostics when using honey, hive debris, or samples of adult bees. The reliability of the conventional PCR was 100% at 6.7 × 10^3^ Colony Forming Unit of *M. plutonius* in 1 g of debris. The sensitivity of the method for the sampled honey, hive debris, and adult bees was 0.867, 0.714, and 1.000, respectively. The specificity for the tested matrices was 0.842, 0.800, and 0.833. The predictive values for the positive tests from selected populations with 52% prevalence were 0.813, 0.833, and 0.842, and the real accuracies were 0.853, 0.750, and 0.912, for the honey, hive debris, and adult bees, respectively. It was concluded that hive debris can effectively be utilized to non-invasively monitor EFB in honey bee colonies.

## 1. Introduction

Honey bees suffer from many pests and pathogens that can weaken or lead to the collapse of their colonies [1]. American foulbrood (AFB, infectious agent *Paenibacillus larvae*) and European foulbrood (EFB, infectious agent Gram-positive bacterium *Melissococcus plutonius*) are the main bacterial infections, and they affect honey bee health by impairing their larval development [2,3]. Both diseases can be detected from the clinical symptoms in infected colonies [4,5]. AFB typically results in a brownish semi-fluid “ropy-mass” [6], while younger EFB-infected larvae are mostly transparent, with visible trachea, and the older larvae die twisted around the cell walls [2]. European foulbrood (EFB) is a globally distributed and economically important disease of honey bees [7]. The bacterium only affects bee larvae shortly after they have hatched via contaminated food. Adult honey bees are not affected but transfer vegetative bacteria, and thus, spread the disease [2,8]. The bacterium multiplies in the digestive tract of the infected larvae after ingestion [9], and they may die at any time between the 4th day and pupation [6]. Whether or not the infection is lethal to all larvae depends on its extent [4,9,10] and how virulent the bacterial strain is [11,12,13,14]. The molecular mechanisms of EFB infection are not currently understood [2,15]. Weakened or dead larvae are attacked by secondary accompanying bacterial invaders [6,10,16].

Outbreaks of EFB have been observed in the Czech Republic, United Kingdom, Switzerland, Norway, and other European countries [17,18]. However, EFB is not limited to Europe as it has also been detected in Asia, America, and Africa [17]. Although the disease is spread worldwide, information about *M. plutonius* strains and their distribution is limited. At present, three different clonal complex (CC) groups that contain strains with several different sequence types have been identified: CC3, CC12, and CC13 [15]. They differ in biochemical characteristics, virulence, and prevalence [19].

One of the basic diagnostic methods for EFB is microscopic detection of *M. plutonius* from carbol fuchsin stained smears of the dead or suspected larvae, but it does not discriminate the secondary invaders [4,20]. Microbial cultivation techniques for *M. plutonius* are slow with variable results because of the contaminating bacteria [20]. More recently, the MALDI-TOF diagnosis technique has been used for AFB [21] and EFB, but it requires culturing on a medium beforehand, which makes it a less attractive option for routine diagnostics (Dainat B. & Schäfer M., personal communication).

Polymerase chain reaction (PCR) based diagnostics sample adult bee tissues, larvae, honey, and pollen with primer pairs specific to *M. plutonius* [20,22]. Enzyme Linked Immunosorbent Assays (ELISA) are used in laboratories to detect antibodies, antigens, and proteins. Polachova et al. [23] recently developed a highly sensitive up-conversion-linked immunosorbent assay (ULISA) that employs photon-up-conversion nanoparticles (UCNPs) for the detection of *M. plutonius* using polyclonal antibodies made in house. Biosensors have also recently been established in laboratory diagnostics [24,25]. The routine use of immune-based techniques is limited, however, because they require EFB specific antibodies. To the best of our knowledge, there are no commercial antibodies targeted to *M. plutonius* epitopes that are currently available (December 2020).

Lateral flow immunoassays are commercially available for field EFB confirmation at apiaries [26]. They function similarly to at-home pregnancy tests, and are useful for distinguishing between EFB symptoms and other diseases with commutable clinical signs, such as AFB, viruses, chalkbrood, and non-pathogenic brood sepsis.

The efficient management of honey bee pests and pathogens is integral for beekeeping practices to help limit the spread of disease by migratory beekeepers, and for sustainable pollination service [27,28]. Antibiotic treatment is banned in many countries; thus, the main control methods are stamping out diseased colonies or the shook swarm method [29]. The spread of honey bee diseases is increased if the colony density is high in particular areas. This seems to be a factor for the transmission of EFB via adult bees that rob or drift between colonies [30,31]. The immediate reaction after finding a new outbreak is important to avoid the spread of the pathogen. In countries where EFB is classified as a notifiable disease, the following is a recommended practice when EFB is encountered in a colony. All colonies at the same apiary and at neighboring apiaries must be inspected: colonies are opened, the brood nest is checked and brood samples with suspected larvae are moved into laboratories for further confirmation. This is time consuming, demanding on human resources, and increases the costs for the prevention of honey bee diseases. Hive debris has been successfully used for AFB spore detection using cultivation [32] and PCR techniques [33,34,35], and for small hive beetle (*Aethina tumida*) using PCR [36,37].

Here we tested the hypothesis that hive debris was suitable for EFB diagnostics using conventional PCR, which has not been done previously. Hive debris can be easily collected over several days (summer) or weeks (winter) using hive bottom boards, which are then sent to a laboratory for *M. plutonius* diagnosis. We optimized a protocol for DNA extraction from hive debris and tested our approach on Swiss EFB positive and negative colonies. This investigation aimed to increase the speed of early EFB diagnosis in wide areas, where hive debris could be easily collected by beekeepers. Furthermore, this would enable prophylactic measures to be implemented earlier, and consequently, earlier eradication could reduce the economic impacts on beekeepers.

## 2. Materials and Methods

### 2.1. Cultivation of Bacterial Isolates

A reference strain of *M. plutonius* CCM 3707^T^ was obtained from Czech Collection of Microorganisms (Masaryk University, Brno, Czech Republic). The strain was inoculated on Basal medium [20] and incubated under anaerobic condition using Genbox anaer (Biomérieux, Marcy I’Etoile, France) at 37 °C for 7 days. Identity of grown colonies was confirmed by PCR [38]. This strain was used for validation of PCR method (see below).

### 2.2. Spiked Debris and Honey for PCR Method Validation

The bacterial colonies of the reference strain *M. plutonius* (CCM 3707^T^) grown up on Basal medium [20] were re-suspended in 0.9% NaCl to an optical density 1 McFarland (3 × 10^8^ CFU/mL). A dilution series of four concentrations of was prepared in 0.9% NaCl 3 × 10^7^ CFU/mL, 3 × 10^6^ CFU/mL, 3 × 10^5^ CFU/mL, or 3 × 10^4^ CFU/mL. A 0.5 mL aliquot of each bacterial suspension was used for dissolving in 4.5 g of debris or honey. Both matrices were tested for *M. plutonius* absence prior to spiking by culturing. Samples were stored at −30 °C until use. Then 1 g of spiked debris or honey was processed for DNA isolation.

### 2.3. Collecting Honey, Debris, and Bees from Colonies

**Figure 1:**
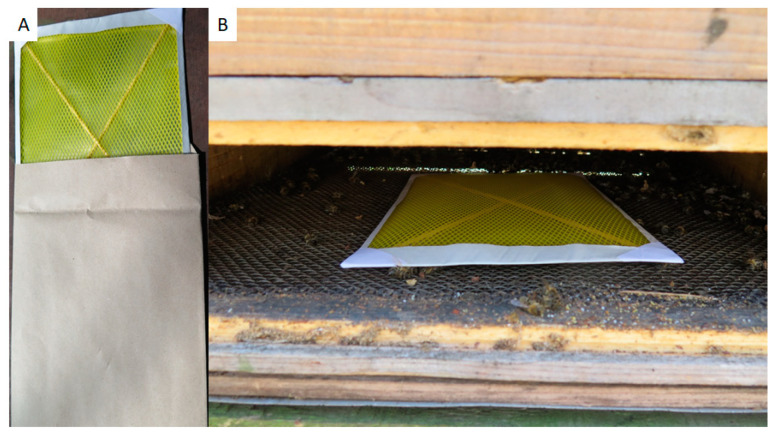
Paper sheets for hive debris collection (**A**); installed in the hive bottom board (**B**).

Field samples were collected in Switzerland, near Bern, in May–June 2018 and 2019. All colonies involved in this study were clinically inspected by trained bee inspectors. Honey was collected from individual colonies directly from the honey chamber. A pool of approx. 200 bees was sampled from a brood chamber of each tested colony, transported to laboratory, and frozen as soon as possible. Hive debris was gathered on bottom boards from individual colonies after 7 days of collection. For easier collection, paper sheets covered with a plastic sieve (common A4 format, supplier Bee Research Institute Dol, Czech Republic) were inserted to the hive bottom for one week (Figure 1). Dry debris was collected and stored in a freezer at −20 °C prior to use. Debris samples with molds are unusable for DNA isolation and *M. plutonius* detection by culture and, therefore, were wasted.

### 2.4. Isolation of DNA from Hive Debris

An amount of 1 g of hive debris was extracted to GITC buffer (5.25 M guanidine isothiocyanate, 50 mM TRIS-HCl, pH 6.4, 20 mM EDTA, 1.3% Triton X-100; autoclaved 120 °C for 20 min, then added β-mercaptoethanol to final 1% concentration) [39]. The mixture was vortexed 3 times for 50 s and then centrifuged at 10,000× *g* for 3 min at room temperature. The supernatant was used for DNA isolation using the DNeasy Plant Mini Kit (Qiagen) according to instruction with minor modifications. Briefly: volume of 400 µL of a debris supernatant was mixed with 130 µL of AP2 buffer. RNase A was not added to the reaction. The samples were mixed and incubated for 5 min on ice. After that, the samples were centrifuged for 5 min at 20,000× *g* and the lysates were pipetted into QIAshredder spin columns. They were centrifuged for 2 min at 20,000× *g* and the flow-through was gently mixed with 1.5 amount of AP3/E buffer. The 650 µL of samples were put into DNeasy Mini spin columns and the columns were centrifuged for 1 min at 6000× *g*. The step was repeated until the whole volume of samples was used. The columns were washed twice with 500 µL of AW buffer and then centrifuged at the highest speed (2 × 2 min at 20,000× *g*). The columns were transferred into new tubes and 50 µL of AE buffer was added. After 5 min of incubation, the DNA was eluted with centrifugation at room temperature (1 min at 6000× *g*).

### 2.5. Isolation of DNA from Honey

An amount of 1 g of honey was fully dissolved with 2.25 mL of sterile distilled water and centrifuged at 16,000× *g* for 30 min at room temperature. The supernatant was discarded and the pellet was gently (by pipetting) dissolved with 1 mL of sterile distilled water and centrifuged again at 16,000× *g* for 30 min at room temperature. The supernatant was discarded and the pellet was used for the DNA isolation. The DNA was isolated from the pellets using the DNeasy Plant Mini Kit (Qiagen, Hilden, Germany) following the manufacturer instructions. Purified DNA was eluted to 50 µL of AE buffer (supplied within the kit).

### 2.6. Isolation of DNA from Honey Bee Workers

The DNA extraction was done according to Roetschi et al. [40]. Bees (12 g = ∼100 workers) were placed in a bag with 50 mL sterile water and crushed twice for 2 min in a stomacher apparatus at high speed. The homogenate was poured into a 50 mL plastic tube and centrifuged for 15 min at 1150× *g*. The supernatant (1.5 mL) was transferred to a 2 mL tube and centrifuged at 20,000× *g* for 2 min. The supernatant was then discarded and the pellet was resuspended in lysis buffer (20 mM Tris-HCl, pH 8.0, 2 mM EDTA, 1.2% Triton X-100, Sigma-Aldrich, St. Louis, MO, USA), amended with 20 mg/mL lysozyme and incubated for one hour at 37 °C. DNA was extracted from the lysate with the EZ1 DNA Tissue kit and a BioRobot EZ1 workstation (Qiagen, Hilden, Germany). Two μL of the extracted DNA was used as the template for real-time PCR.

### 2.7. Polymerase Chain Reaction (PCR)

Prior to PCR, the concentration and quality of isolated DNA from samples were quantified by a Synergy HT microplate reader (BioTek, Bad Friedrichshall, Germany). FastStart Taq DNA Polymerase (Roche, Basel, Switzerland) was used for PCR in a final volume of 25 µL; for the details, see Supplementary Material File S1. When a PCR product of 16S rRNA was detected on the gel, the reaction was judged as positive results. The length of the EFB-specific PCR product was in accordance with the previously published data: 831 bp [38]. The amplified fragments were analyzed by Sanger sequencing using commercial service and subsequently queried in BLAST (NCBI) to confirm identity and coverage of the PCR product and the template sequence of the *M. plutonius* 16S rRNA gene. BioEdit software [41] was used as a tool for visualization of the Sanger sequencing results.

### 2.8. qPCR from Adult Honey Bees

To determine the presence of *M. plutonius* in the extract of adult workers, we used the triplex qPCR described by Dainat et al. [42]. This triplex real-time PCR allows us to analyze the following target organisms: *M. plutonius*, *P. larvae*, and *Apis mellifera*. The PCR was set up in a 20 μL volume; for details, see Supplementary Material File S1.

### 2.9. Statistics

The sensitivity is the ability to recognize a truly positive samples (laboratory result is positive and the colony shows clinical symptoms). Specificity means the ability to recognize a truly negative sample (laboratory result is negative and the colony shows no clinical symptoms). Predictive value of a positive test shows the probability of the presence of disease if the laboratory test is positive. Predictive value of a negative test is the probability that the colony is healthy if the laboratory test is negative. Predictive value of positive and negative tests involves the prevalence of the disease in the studied population, where prevalence is the ratio of clinically positive items from the total population [43,44].

$$\mathrm{Predictive}\;\mathrm{value}\;\mathrm{of}\;\mathrm{positive}\;\mathrm{test}\;\mathrm{based}\;\mathrm{on}\;\mathrm{population}\;\mathrm{level}\;\;\;=\;\left(\frac{\mathrm{sensitivity}\;\times \mathrm{prevalence}}{\mathrm{senstivity}\;\times \mathrm{prevalcene}\;\left(1-\mathrm{specificity}\right)\times \left(1-\mathrm{prevalence}\right)}\right)$$

We adopted the concept of combined sensitivity and specificity [45,46] according to the standard equation:

$$\begin{array}{l} \mathrm{Real}\;\mathrm{Accuracy}\\ =\;\left(\frac{\#\;\mathrm{of}\;\mathrm{true}\;\mathrm{positives}\;+\;\#\;\mathrm{of}\;\mathrm{true}\;\mathrm{negatives}\;}{\#\;\mathrm{of}\;\mathrm{true}\;\mathrm{positives}\;+\;\#\;\mathrm{of}\;\mathrm{true}\;\mathrm{negatives}\;+\;\#\;\mathrm{of}\;\mathrm{false}\;\mathrm{negatives}\;+\;\#\;\mathrm{of}\;\mathrm{false}\;\mathrm{positives}}\right) \end{array}$$

where true positives are colonies showing EFB symptoms that tested positive for the disease, true negatives are healthy colonies that tested negative, false negatives are colonies showing EFB symptoms that tested negative, and false positives are healthy colonies that tested positive for the EFB disease.

To better understand missing data values in Real Accuracy, we calculate Pessimistic Accuracy that represents a “worst-case scenario” that also incorporates colonies with unknown symptoms and/or unknown EFB test information. The worst-case scenario accuracy is calculated according to the following equation:

$$\mathrm{Pessimistic}\;\mathrm{Accuracy}=\;\left(\frac{\#\;\mathrm{of}\;\mathrm{healthy}\;\mathrm{colonies}\;\mathrm{with}\;\mathrm{negative}\;\mathrm{test}+\#\;\mathrm{of}\;\mathrm{positively}\;\mathrm{tested}\;\mathrm{colonies}\;\mathrm{with}\;\mathrm{EFB}\;\mathrm{symptoms}}{\mathrm{total}\;\#\;\mathrm{of}\;\mathrm{colonies}}\right)$$

In this work, we use Average Accuracy to compare varying quality and size data sets and Combined Accuracy Pessimistic to evaluate missing info value. An important measure of disease spread in the tested population is EFB prevalence that was calculated based on the ratio of positive and negative colonies inspected for EFB.

## 3. Results

### 3.1. Primer Specificity

The primers used to amplify the *M. plutonius* template sequence were verified using the PCR conditions described in Material and Methods. We confirmed 100% identity and 100% coverage of the 16S rRNA gene from the *M. plutonius* NCDO 2443 (synonymous of LME 9003 strain) strain, which is in accordance with previous research [38]. Supplementary Material File S1 describes the identity of the sequences and further compares the 16S rRNA gene among other *M. plutonius* strains. It should be noted that the primers can be utilized to detect all known *M. plutonius* strains as they cover highly conserved regions where the strains are 100% identical. We then tested whether these primers could also amplify the 16S rRNA gene of the other microbes, mainly the second infection candidates of the genus *Enterococcus* and *Lactobacillus* [6,46], when using the same PCR conditions. The results are presented in Supplementary Material File S1.

### 3.2. Reliability of PCR Detection from Honey and Hive Debris

The reliability of the method was tested using six sets of honey and debris samples with four different CFUs of *M. plutonius* (6.7 × 10^2^; 10^3^; 10^4^, and 10^5^ CFU per 1 g of honey or debris). Detection success with 6.7 × 10^2^ CFU of *M. plutonius* in 1 g of hive debris and 1 g of honey was 50% and 80%, respectively. When there were more than 6.7 × 10^3^ CFUs, there was 100% reliability of the *M. plutonius* detection using conventional PCR (Table 1).

### 3.3. Comparison of PCR Confirmations Using Different Sample Types

Inspected colonies were assumed to be EFB positive when clinical symptoms were present. Positivity of the result was based on a positive PCR or qPCR reaction and the presence of clinical symptoms. The results were positive for EFB in 38.2%, 41.7%, and 47.1% of the honey, debris, and adult bee samples, respectively (Table 2). In total, 10 honey samples, 8 debris samples, and 8 bee samples were missing or degraded and 2 of the adult bee samples tested using qPCR were confirmed as unsure, because the Ct value was over 30, and detailed results are listed in Supplementary Material File S2.

### 3.4. Sensitivity and Specificity

Based on our results from hypothetical populations with varying disease prevalence, we performed a comparative analysis of the statistical parameters on a testing population with 52% prevalence of the disease (Table 3). The specificity of the tests was comparable for both methods and all three samples were analyzed, whereas sensitivity was 100% only for the adult bees with the qPCR. Although the sensitivity of the conventional PCR when the hive debris was used as the template was only 70% in the testing population, predictive values for the positive tests were comparable to the other two sample groups.

### 3.5. Predictive Values of the Positive and Negative Tests Based on the Prevalence of M. plutonius in Hypothetical Populations

When there was a high prevalence of *M. plutonius* in a population, the predictive value of the positive test was relatively high when compared to the populations with a low prevalence of the disease, regardless of the sample type.

The highest predictive value for the positive test was 0.934 for the adults when the *M. plutonius* prevalence was 30%, and 0.006 when prevalence was 0.1%. In contrast, the predictive value of the negative test increased with the decreasing prevalence of *M. plutonius* in the tested population; however, this value remains high even for populations with high levels of prevalence for *M. plutonius* (Table 4).

## 4. Discussion

Hive debris may indicate the health status of honey bee colonies and can be easily collected by beekeepers [32,33,34,35,36]. It is common and an easy task to sample honey, suspected broods, or adult bees [20]; however, this is time consuming, dependent on weather conditions, requires opening colonies, and increases the risk of losing the queen during the sampling process and requires significant logistics for sending the samples to the laboratory.

The optimized method for DNA isolation presented here allows for large amounts of hive debris to be processed using an in-house made GITC buffer with DNaeasy Plant Mini Kits (Qiagen). Ryba, Titera, Haklova, and Stopka [33] reported that PCR could confirm the presence of *P. larvae* spores (AFB) from hive debris at levels of 10^2^ spores per 1 g of debris. The reliability of the PCR results in this investigation were 50% at 6.7 × 10^2^ CFU of *M. plutonius* per 1 g of debris. Reports from Alippi et al. [47] and Ryba, Titera, Haklova, and Stopka [33] also showed that there were detection limits of 283 and 10^5^ spores of *P. larvae* per 1 g of honey, respectively. This study detected 80% reliability for the detection of *M. plutonius* at 6.7 × 10^2^ CFU per 1 g honey, and this is comparable to the results for the *P. larvae* spores. For comparison, periods of debris collection on the bottom board [34], the place the honey was sampled from [48], and the extraction methods could all influence the reliability of the PCR confirmation of AFB from hive debris or honey, as previously described by Forsgren and Laugen [49].

To evaluate the detection of EFB when using conventional or qPCR methods on three of the most easily accessible hive samples, we selected several statistical measures of the test performance. Here, sensitivity and specificity were calculated based on the clinical symptoms detected by the bee inspector, which is considered a “golden standard”. Predictive values of positive and negative tests that were based on the prevalence of a disease among different geographically isolated populations were also an important measure of disease spread. Besides the predictive values, real accuracy was a strict measure of test performance and, thus, more appropriate for the comparison of data sets. Pessimistic accuracy counts occurred for the missing data in cases where a test underperformed, i.e., pessimistic accuracy shows uncertainty. There are undoubtedly many other statistical parameters that could be used to compare the testing methods [50,51]; here, we suggest routine calculations of the above-mentioned statistical parameters.

Bee inspectors collected samples from the suspected colonies, but all sample types were not always available, because of beekeeper’s intervention or technical reasons.

Here, the positive hive debris and honey samples were detected by either conventional PCR or qPCR and the results compared. The methods differed in the time required for analysis, price, and sensitivity, but both are used in clinical routines [52,53]. Colonies with positive adult bees that were detected using qPCR were not detected as positive from conventional PCR from the honey (1 case), hive debris (4 cases), or bee inspector (3 cases; for further details see Supplementary Material File S2). Adult bees can carry and disperse *M. plutonius*. Colonies in apiaries without EFB positive colonies that were close to EFB positive apiaries also had 30% positive detections for *M. plutonius* [54]. Social immunity plays an important role in removing diseased or dead brood from the hive nest [55,56]. Colonies with high levels of hygienic behavior could decrease the infectious pressure by removing the diseased broods [57,58,59], and thus, EFB would remain undetected by the bee inspector. This might be the cause of the missing clinical symptoms of colonies 27, 29, and 34 (see Supplementary Material File S2). Those colonies were confirmed in the laboratory as positive from debris, honey, and bee samples. The colonies were not tested or inspected later in this study. False-positive samples are also informative from a practical point of view. Those colonies might be sources for the agents (infection) and should be monitored, put under regimented controls (no exchange of frames between colonies, no moving), or preventive prophylactic measures should be performed (shook swarm method or replace old frames; [29]. In general, a colony diagnosed in the laboratory as positive but without clinical symptoms could be at risk of future disease in the apiary.

Paper sheets allow for the collection of hive debris without the need for further processing at the apiary. A beekeeper simply places the paper on the bottom board and collects hive debris for the recommended time. Here, we used 1 week during early spring time. Exposure time could be extended during the cold weather period because there is reduced activity in the colonies. The paper sheets could then be sent to the laboratory to confirm a colony at an apiary level or as a pooled sample. Pooled samples were not tested in this study, as they could be validated in another assay. All these steps could be done by beekeeper; hence, apiaries from a wide region could be sampled in a very short time with very low costs. Bee inspectors could then visit apiaries in a more targeted manner at a later date, based on the positive results from the laboratory analysis, thus reducing the time and expense required for the visual inspection. Saving time and spotting infected apiaries early in the season is important to quickly prevent the spread of disease. An example of a hypothetical schedule for sampling and testing in suspected regions is as follows: beekeepers collect hive debris, the laboratory make confirmation of hive debris by PCR, and then at an early stage of infection, a bee inspector could inspect the suspected apiary or colonies. Honey and bee samples appeared to provide more reliable results. In contrast to previous findings, the honey collected from the brood chambers of the EFB positive colonies were confirmed as positive in only 35% [48]. In general, the collection of honey, broods, or adults is more invasive and difficult to carry out early in the season and, therefore, more difficult to utilize in practice.

## 5. Conclusions

Based on our results, while the qPCR detection from the adult bees displayed higher sensitivity, the conventional PCR could be an appropriate alternative. Harvesting honey and sampling adult bees in brood nests is invasive for colonies and limited to the active season. Our findings support the idea that hive debris is a valuable matrix to utilize for *M. plutonius* detection with only a slight reduction in the sensitivity and specificity, in comparison with other tested materials. Sampling hive debris during the winter could be a preliminary check that could help to target the future in-person inspections.

## Figures and Tables

**Table 1:** Reliability of the analysis using Colony Forming Units (CFUs) of *M. plutonius* in spiked samples.

Technical Replicate	CFU *M. plutonius* in 1 g Debris					CFU *M. plutonius* in 1 g Honey				
	0.00 × 10^0^	6.70 × 10^2^	6.70 × 10^3^	6.70 × 10^4^	6.70 × 10^5^	0.00 × 10^0^	6.70 × 10^2^	6.70 × 10^3^	6.70 × 10^4^	6.70 × 10^5^
1	negative	positive	positive	positive	n/a	negative	positive	positive	positive	positive
2	negative	positive	n/a	positive	n/a	negative	positive	positive	positive	positive
3	negative	negative	positive	positive	positive	negative	negative	positive	positive	positive
4	negative	negative	positive	positive	positive	negative	positive	positive	positive	positive
5	negative	positive	positive	positive	positive	negative	positive	positive	positive	positive
6	negative	negative	positive	positive	positive	negative	n/a	n/a	n/a	n/a
Reliability		50%	100%	100%	100%		80%	100%	100%	100%

n/a: not analyzed.

**Table 2:** Overview of the PCR results obtained for each sample matrix normalized to the clinical inspections.

Status	Description	Honey (n)	Honey (% from Total Analyzed)	Debris (n)	Debris (% from Total Analyzed)	Adult Bees (n)	Adult Bees (% from Total Analyzed)
Positive	PCR positive with clinical symptoms	13	38.2	15	41.7	16	47.1
False positive	PCR positive without clinical symptoms	3	8.8	3	8.3	3	8.8
Negative	PCR negative without clinical symptoms	16	47.1	12	33.3	15	44.1
False negative	PCR negative with clinical symptoms	2	5.9	6	16.7	0	0.0

**Table 3:** Comparative statistical parameters in a model population with 52% prevalence of the *M. plutonius* in honey, hive debris and adult bees.

Bayes Statistics	Honey (Conventional PCR)	Hive Debris (Conventional PCR)	Adult Bees (qPCR)
Sensitivity	0.867	0.714	1.000
Specificity	0.842	0.800	0.833
Predictive values of positive test	0.813	0.833	0.842
Predictive value of negative test	0.889	0.667	1.000
Real accuracy	0.853	0.750	0.912
Pessimistic accuracy	0.659	0.614	0.738

**Table 4:** Predictive values for the positive and negative tests based on the prevalence of *M. plutonius* in honey, hive debris, and adult bees.

Hypothetical Prevalence of *M. plutonius*	Honey (Conventional PCR)		Hive Debris (Conventional PCR)		Adult Bees (qPCR)	
	Positive Test	Negative Test	Positive Test	Negative Test	Positive Test	Negative Test
0.3	0.702	0.936	0.605	0.867	0.934	1.000
0.1	0.379	0.983	0.284	0.962	0.400	1.000
0.01	0.053	0.998	0.035	0.996	0.048	1.000
0.001	0.005	1.000	0.004	1.000	0.006	1.000

## Data Availability

Detailed data are available upon request.

## Supplementary Materials

The following are available online at https://www.mdpi.com/2075-4450/12/2/150/s1, Supplementary Material File S1: PCR and primer details; Supplementary Material File S2: Detailed results from individual colonies.
