# Supplementary material for: Melissococcus plutonius Can Be Effectively and Economically Detected Using Hive Debris and Conventional PCR
Source: Insects. 2021 Feb 9;12(2):150. doi: 10.3390/insects12020150 (PMC7916248; doi:10.3390/insects12020150)
Supplement: Supplementary file 1 [file insects-12-00150-s001.zip › S1_Supplementary material.pdf]

Conventional polymerase chain reaction (PCR)

The premix contained following FastStart reagents: 1× PCR buffer without $MgCl_2$, 2.5 mM $MgCl_2$, 1× GC rich solution,, 0.2 mM of each dNTPs, 2.5 U FastStart Taq DNA Polymerase. Primers specific towards the sequence of *M. plutonius* (strain LMG 9003) gene 16S rRNA (GenBank accessions: X75751.1 and X75752.1) were used in 0.6 μM concentrations (Forward primer 5'-GAAGAGGAGTTAAAAGGCGC-3'; Reverse primer 5'-TTATCTCTAAGGCGTTCAAAGG-3') [1]. As a template DNA for PCR, 130 – 220 ng of debris samples or 40 - 80 ng of honey samples. Amplification was performed in a Thermocycler T100 (Bio-Rad, USA) with following parameters: 5 min of initial denaturation at 95 °C, 34 cycles of 95 °C for 1 min, 55 °C for 1 min, 72 °C for 1 min; followed by a final elongation step of 72 °C for 5 min. Ten microliters of the PCR reactions were analyzed by electrophoresis (80 V, 40 min) in 0.8% (w/v) agarose gel. The DNA fragments were stained with GelRed (Biotium, Czech Republic; according to the manufacturer's manual) and visualized by Gel Doc Ez Imager (Bio-Rad, USA). 2-Log DNA Ladder (0.1-10 kbp) (Neb, USA) was used as a DNA marker. The amplified fragments were analysed by Sanger sequencing using commercial service and subsequently queried in BLAST (NCBI) to confirm identity and coverage of the PCR product the template sequence of the *M. plutonius* 16S rRNA gene. BioEdit software [2] was used as a tool for visualization of the Sanger sequencing results.

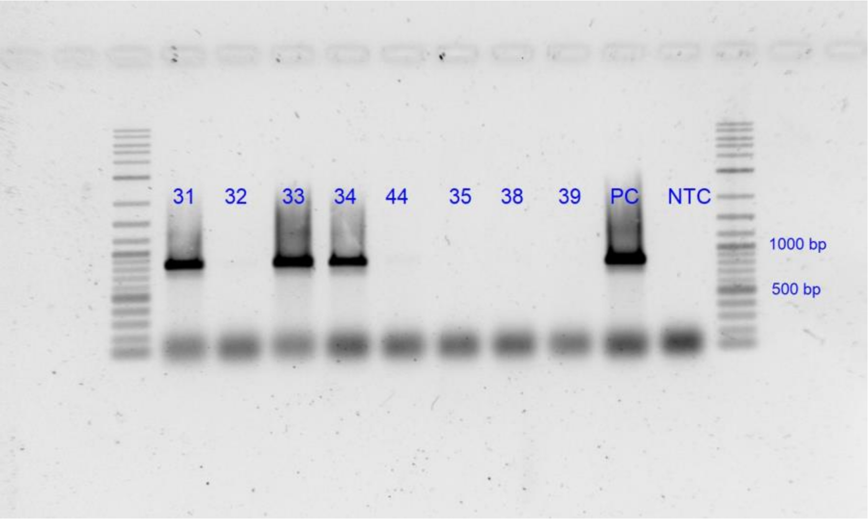

S1 Figure 1: Representative gel electroforesis of conventional PCR products from honey. Numbers – ID of colonies, PC – positive control, NTC – negative control.

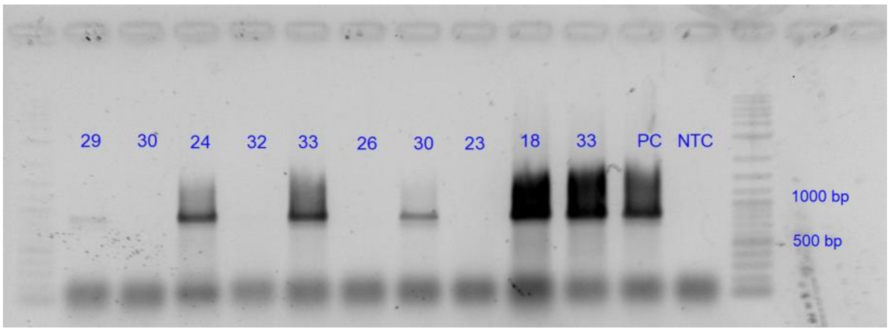

S1 Figure 2: Representative gel electroforesis of conventional PCR products from hive debris. Numbers – ID of colonies, PC – positive control, NTC – negative control.

qPCR from adult honey bees

This triplex real-time PCR allows to analyze the following target organisms: *M. plutonius*, *P. larvae* and *Apis mellifera*. The PCR was set up in a 20 µL volume, 2× Mastermix (SensiFast Probe No-ROX Kit from Bioline) containing 400 nM of each primer, 100 nM of each probe and 5 µl DNA-extract (1 ng/µL). The amplifications were performed with the Rotor-Gene 6000, Qiagen. According to the Bioline recommendations the following cycling program was applied: 5 min of initial denaturation at 95 °C and 45 cycles of 10 s at 95 °C and 30 s at 60 °C. The limit of detection (LOD) for EFB was set at a quantification cycle Cq-value of 30.

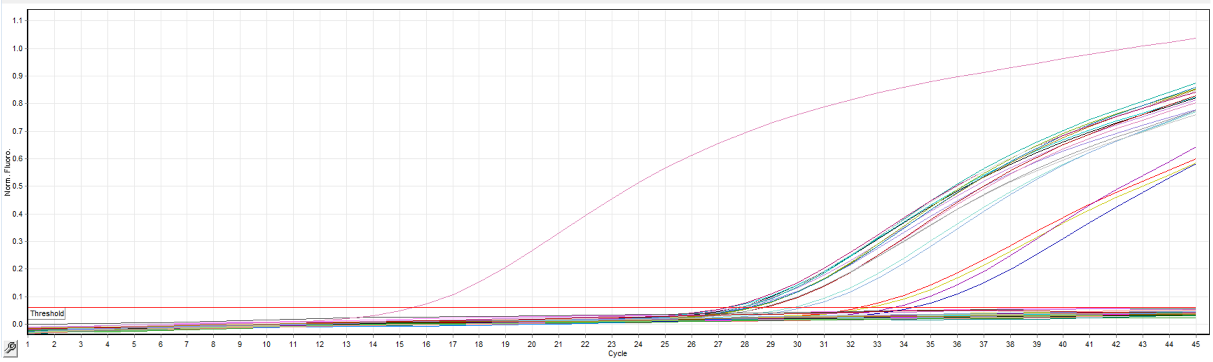

S1 Figure 3: Representative amplification curves of qPCR from adult bees, for the Ct values see below Table S1.

S1 Table S1: Representative qPCR result from adult bees with Ct values and control samples. The colours are in agreement with amplification curves in Figure 3, see above.

No.	Color	Name	Type	Ct	Ct Comment
1		1192 Colony ID 15	Unknown	32.37	
2		1192 Colony ID 15	Unknown	32.79	

No.	Color	Name	Type	Ct	Ct Comment
3		1193 Colony ID 20	Unknown		
4		1193 Colony ID 20	Unknown		
5		1194 Colony ID 21	Unknown		
6		1194 Colony ID 21	Unknown		
7		1195 Colony ID 22	Unknown		
8		1195 Colony ID 22	Unknown		
9		1196 Colony ID 23	Unknown		
10		1196 Colony ID 23	Unknown		
11		1197 Colony ID 25	Unknown	27.75	
12		1197 Colony ID 25	Unknown	27.47	
13		1198 Colony ID 26	Unknown		
14		1198 Colony ID 26	Unknown		
15		1199 Colony ID 27	Unknown	29.85	
16		1199 Colony ID 27	Unknown	30.24	
17		1200 Colony ID 28	Unknown	28.06	
18		1200 Colony ID 28	Unknown	27.72	
19		1201 Colony ID 29	Unknown	28.70	
20		1201 Colony ID 29	Unknown	28.78	
21		1202 Colony ID 30	Unknown	28.26	
22		1202 Colony ID 30	Unknown	28.34	
23		1203 Colony ID 31	Unknown	27.94	
24		1203 Colony ID 31	Unknown	28.22	
25		1204 Colony ID 32	Unknown	34.35	
26		1204 Colony ID 32	Unknown	33.63	
27		1205 Colony ID 33	Unknown	27.35	
28		1205 Colony ID 33	Unknown	27.80	

No.	Color	Name	Type	Ct	Ct Comment
29		1206 Colony ID 34	Unknown	28.72	
30		1206 Colony ID 34	Unknown	28.69	
31		1207 Colony ID 35	Unknown		
32		1207 Colony ID 35	Unknown		
33		1208 Colony ID 36	Unknown		
34		1208 Colony ID 36	Unknown		
35		1209 Colony ID 37	Unknown		
36		1209 Colony ID 37	Unknown		
37		1210 Colony ID 38	Unknown		
38		1210 Colony ID 38	Unknown		
39		1211 Colony ID 39	Unknown		
40		1211 Colony ID 39	Unknown		
41		1212 Colony ID 40	Unknown		
42		1212 Colony ID 40	Unknown		
43		1213 Colony ID 41	Unknown		
44		1213 Colony ID 41	Unknown		
45		1214 Colony ID 42	Unknown		
46		1214 Colony ID 42	Unknown		
47		1215 Colony ID 43	Unknown		
48		1215 Colony ID 43	Unknown		
49		1216 Colony ID 44	Unknown		
50		1216 Colony ID 44	Unknown		
51		c + MP 1ng/ul	Unknown	15.56	
52		c -	Unknown		
53		c pcr	Unknown		

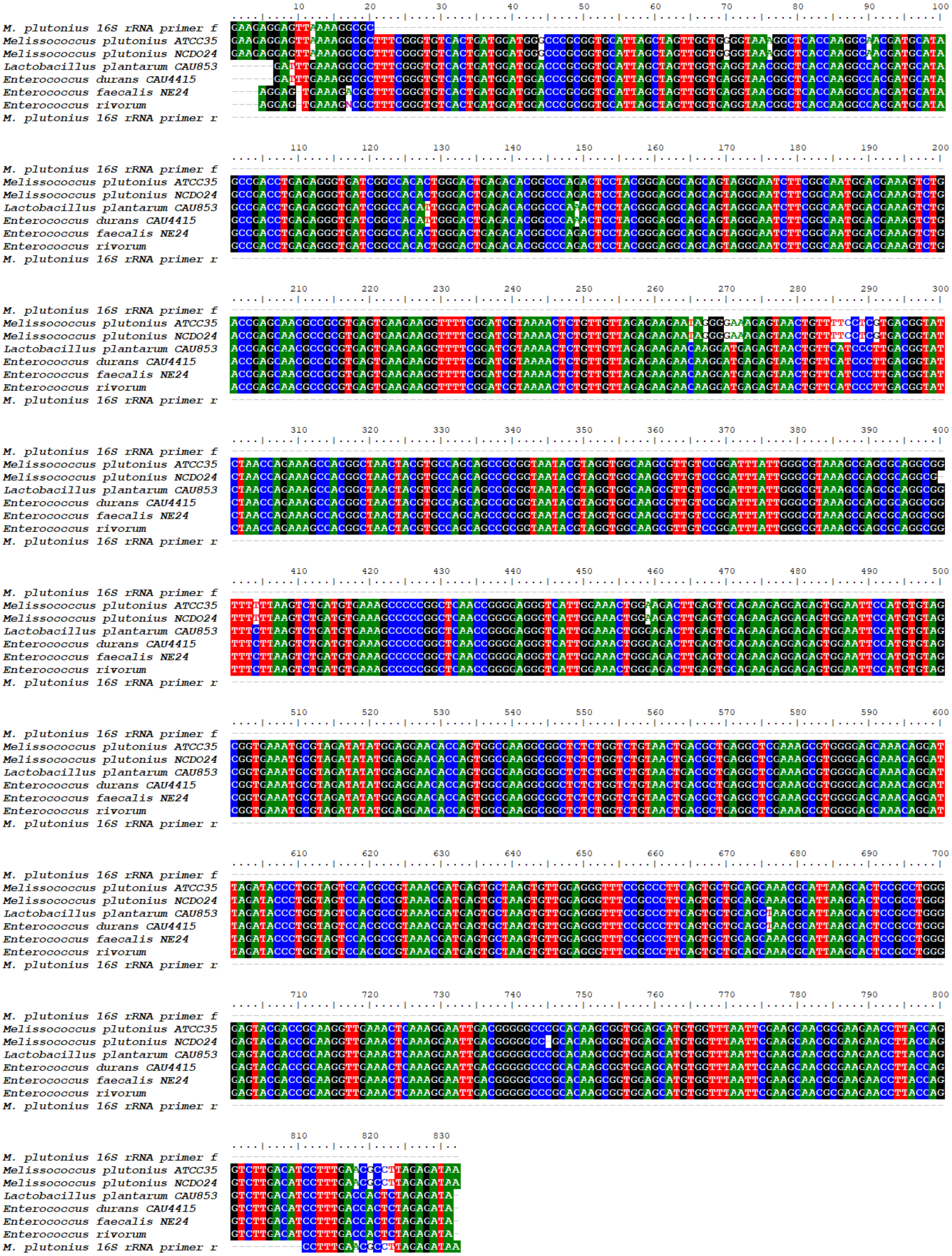

S1 Figure 4 ClustalW multiple alignment of *Melissococcus plutonius* 16S rRNA PCR product amplified with specific primers [1] and potential side targets of the primers - high similarity microbial sequences as derived from NCBI BLASTn.

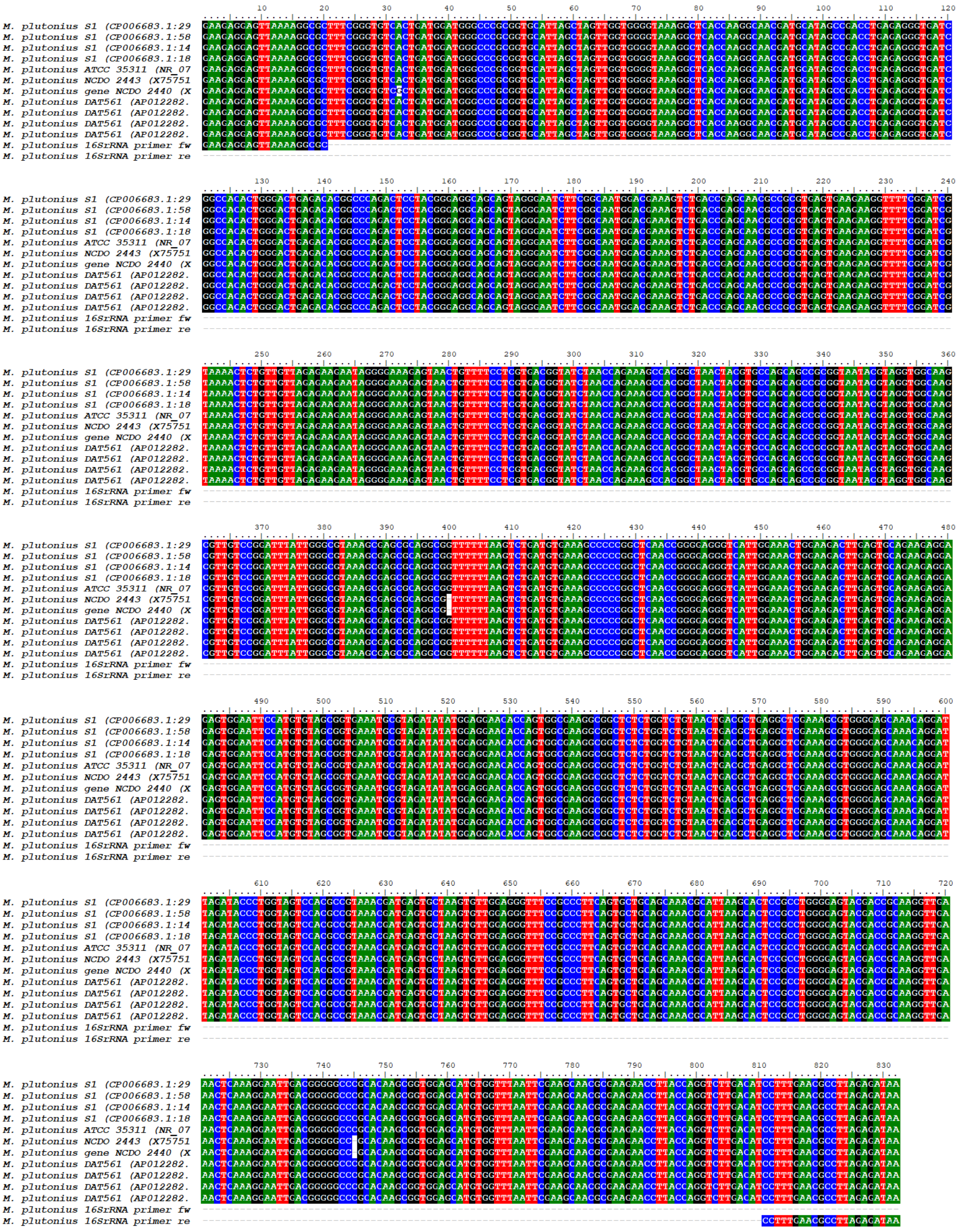

S1 Figure 5. ClustalW multiple alignment of *Melissococcus plutonius* 16S rRNA PCR product amplified with specific primers [1], various whole-genome sequenced strains (S1, ATCC 35311 and DAT561) are shown for comparison to original Sanger sequencing data of NCDO 2440 and NCDO 2443 strains. In two out of four WGS strains more that one locus was identified and therefore included into this alignment.

S1 Table 2: BLASTn sequence identity table for all analyzed accessions.

Microorganism (strain)	GenBank ID	Identity (%)	length (bp)	mismatches	gap opens	query strart	query end	subject start	subject end	e-value	bit score
M. plutonius NCDO 2443	X75751.1	100	830	0	0	1	830	84	913	0	1533
M. plutonius NCDO 2440	X75752.1	99.9	830	1	0	1	830	84	913	0	1528
E. rivorum HAMBI 3120	FR746104.1	97.1	828	19	5	5	829	185	1010	0	1393
E. faecalis NE24	HM244968.1	97.1	828	19	5	5	829	151	976	0	1391
E. rivorum HAMBI 3124	FR746106.1	97.0	828	20	5	5	829	185	1010	0	1387
L. plantarum CAU8538	MF583017.1	96.9	826	22	4	7	829	155	979	0	1378
E. durans CAU4415	MF582894.1	96.9	826	22	4	7	829	133	957	0	1378

References

1. Govan, V.A.; Brozel, V.; Allsopp, M.H.; Davison, S. A pcr detection method for rapid identification of melissococcus pluton in honeybee larvae. *Appl Environ Microbiol* **1998**, *64*, 1983-1985. 10.1128/AEM.64.5.1983-1985.1998.
2. Hall, T.A. Bioedit : A user-friendly biological sequence alignment editor and analysis program for windows 95/98/nt. *Nucleic Acids Symp. Ser.* **1999**, *41*, 95-98.
