# Supplementary material for: Melissococcus plutonius Can Be Effectively and Economically Detected Using Hive Debris and Conventional PCR
Source: Insects. 2021 Feb 9;12(2):150. doi: 10.3390/insects12020150 (PMC7916248; doi:10.3390/insects12020150)
Supplement: Supplementary file 1 [file insects-12-00150-s001.zip › S2_Supplementary material.pdf]

Table S2: Characterization of colonies used in this study. Clinical inspections of colonies and PCR diagnosis of the samples. Symbols: "+" (red): positive; "-" (blue): negative; "?" (orange): uncertainty; "n/a" (yellow): not analyzed.

Location	Colony ID	Collecting debris (day, month, year)	Clinical symptoms (inspection)	Honey (endpoint PCR)	Hive debris (endpoint PCR)	Adult bees (qPCR)
Krauchthal	1	31. 5. - 8. 6. 2018	+	+	+	n/a
Bellechasse	2	31. 5. - 8. 6. 2018	+	n/a	+	+
Bellechasse	3	31. 5. - 8. 6. 2018	+	n/a	-	+
Bellechasse	4	31. 5. - 8. 6. 2018	+	+	+	+
Bellechasse	5	31. 5. - 8. 6. 2018	+	+	+	+
Bellechasse	6	31. 5. - 8. 6. 2018	+	-	-	+
Bellechasse	7	31. 5. - 8. 6. 2018	+	+	n/a	+
Bellechasse	8	31. 5. - 8. 6. 2018	+	+	+	+
Bellechasse	9	31. 5. - 8. 6. 2018	+	n/a	-	+
Bellechasse	10	31. 5. - 8. 6. 2018	+	+	+	+
Bellechasse	11	31. 5. - 8. 6. 2018	+	+	-	n/a
Bellechasse	12	31. 5. - 8. 6. 2018	+	+	n/a	+
Bellechasse	13	31. 5. - 8. 6. 2018	+	n/a	-	+
Bellechasse	14	31. 5. - 8. 6. 2018	+	+	+	n/a
Noflen 1	15	17. 5. - 28. 5. 2019	+	-	+	?
Noflen 1	16	17. 5. - 28. 5. 2019	+	n/a	+	n/a
Noflen 1	17	17. 5. - 28. 5. 2019	+	n/a	-	n/a
Noflen 1	18	17. 5. - 28. 5. 2019	+	n/a	+	n/a
Noflen 1	19	17. 5. - 28. 5. 2019	-	n/a	-	n/a
Noflen 1	20	17. 5. - 28. 5. 2019	-	-	-	-
Noflen 1	21	17. 5. - 28. 5. 2019	-	-	n/a	-
Noflen 1	22	17. 5. - 28. 5. 2019	-	-	-	-
Noflen 1	23	17. 5. - 28. 5. 2019	-	-	-	-
Noflen 1	24	17. 5. - 28. 5. 2019	-	n/a	-	n/a
Noflen 2	25	29. 5. - 4. 6. 2019	+	+	+	+
Noflen 2	26	29. 5. - 4. 6. 2019	-	-	-	-
Noflen 2	27	29. 5. - 4. 6. 2019	-	+	+	+
Noflen 2	28	29. 5. - 4. 6. 2019	+	+	+	+
Noflen 2	29	29. 5. - 4. 6. 2019	-	+	+	+
Noflen 2	30	29. 5. - 4. 6. 2019	+	n/a	+	+
Noflen 2	31	29. 5. - 4. 6. 2019	+	+	+	+
Noflen 2	32	29. 5. - 4. 6. 2019	-	-	-	?
Noflen 2	33	29. 5. - 4. 6. 2019	+	+	+	+
Noflen 2	34	29. 5. - 4. 6. 2019	-	+	+	+
Liebefeld	35	5. 5. - 14. 6. 2019	-	-	n/a	-
Liebefeld	36	5. 5. - 14. 6. 2019	-	-	n/a	-
Liebefeld	37	5. 5. - 14. 6. 2019	-	-	n/a	-
Liebefeld	38	5. 5. - 14. 6. 2019	-	-	-	-
Liebefeld	39	5. 5. - 14. 6. 2019	-	-	-	-

Liebefeld	40	5. 5. - 14. 6. 2019	-	-	-	-
Liebefeld	41	5. 5. - 14. 6. 2019	-	-	-	-
Liebefeld	42	5. 5. - 14. 6. 2019	-	-	n/a	-
Liebefeld	43	5. 5. - 14. 6. 2019	-	-	-	-
Liebefeld	44	5. 5. - 14. 6. 2019	-	-	n/a	-

Some samples were not analysed (n/a) because of several technical reasons, which include: the shook swarm method was performed (e.g. colony ID 19, 24), no honey was present (e.g. colony 30), stamping out of diseased colonies (e.g. colony 16, 17, 18), or sample degradation (mould, fermentation, wax moths).
